# Supplementary material for: Highly accurate long-read HiFi sequencing data for five complex genomes
Source: Sci Data. 2020 Nov 17;7:399. doi: 10.1038/s41597-020-00743-4 (PMC7673114; doi:10.1038/s41597-020-00743-4)
Supplement: Supplementary file 1 — Supplementary Figures and Tables [file 41597_2020_743_MOESM1_ESM.pdf]

Table of Contents

Supplementary Figure 1 .... S-2
Supplementary Figure 2 .... S-3
Supplementary Figure 3 .... S-4
Supplementary Figure 4 .... S-5
Supplementary Figure 5 .... S-6
Supplementary Table 1 .... S-7
Supplementary Table 2 .... S-8
Supplementary Table 3 .... S-9

Supplementary Figures

Supplementary Figure 1

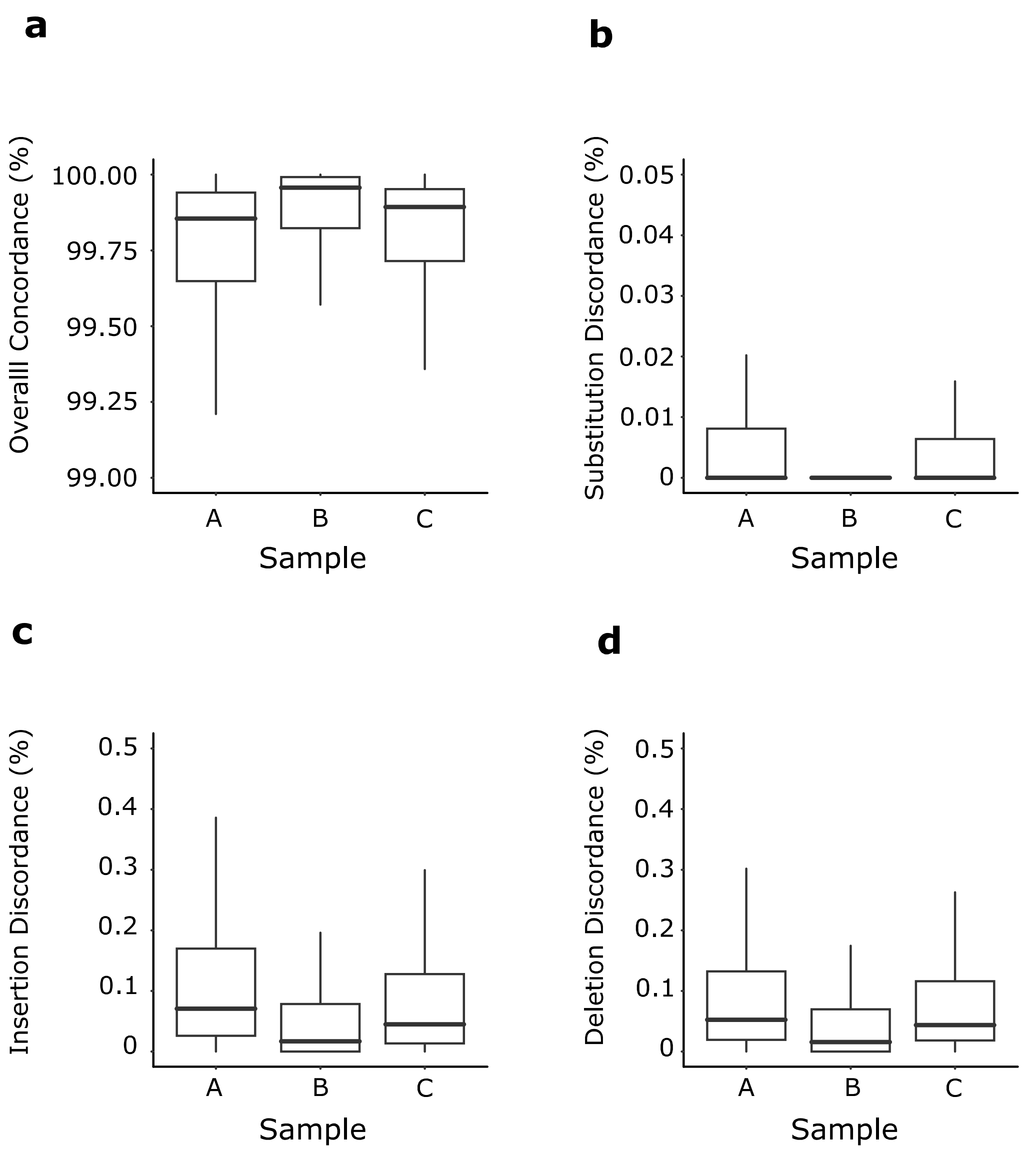

Supplementary Figure 1. Concordance and breakdown of discordances of HiFi reads for three samples with high quality references. (A*: Z. mays B73*: B: Mock Metagenome C: *M. musculus C57BL/6J*) (a) Total concordance with the references listed in **Table 1** and **Supplementary Table 1**. (b) mismatch discordances (c) Insertions discordances (d) deletion discordances. Boxes extend from lower to upper quartiles; the median is represented by the short line within the box; whiskers extend 1.5 interquartile distances.

Supplementary Figure 2

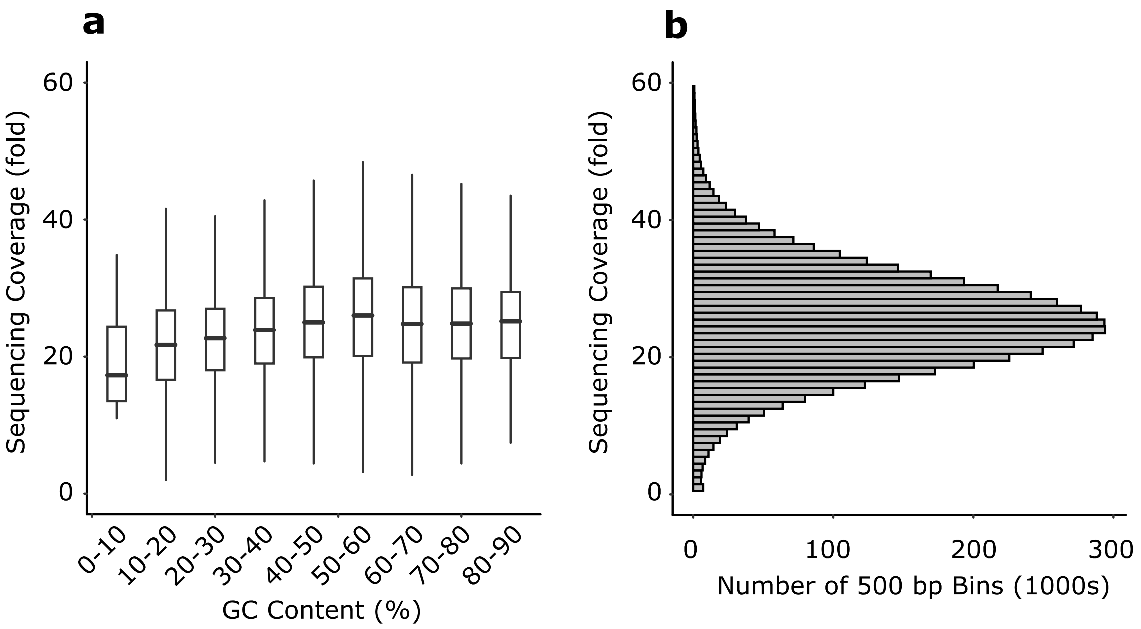

Supplementary Figure 2. HiFi Coverage Uniformity for the *M. musculus* C57BL/6J genome (GRCm38.p6) a) Coverage distribution levels across genomic region windows (500 bp) of varying GC content. b) distribution of whole genome coverage in 500 bp windows. Boxes extend from lower to upper quartiles; the median is represented by the short line within the box; whiskers extend 1.5 interquartile distances.

Supplementary Figure 3

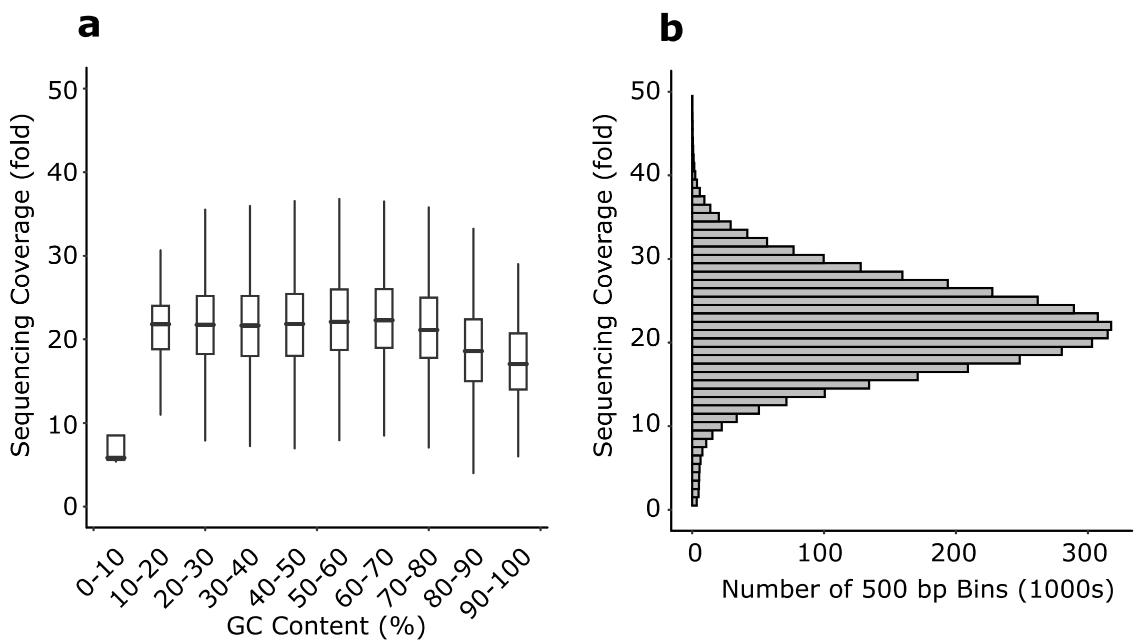

Supplementary Figure 3. HiFi Coverage Uniformity for the *Z. mays* B73 genome (Zm-B73-REFERENCE-NAM-5.0) a) Coverage distribution levels across genomic region windows (500 bp) of varying GC content. b) distribution of whole genome coverage in 500 bp. Boxes extend from lower to upper quartiles; the median is represented by the short line within the box; whiskers extend 1.5 interquartile distances.

Supplementary Figure 4

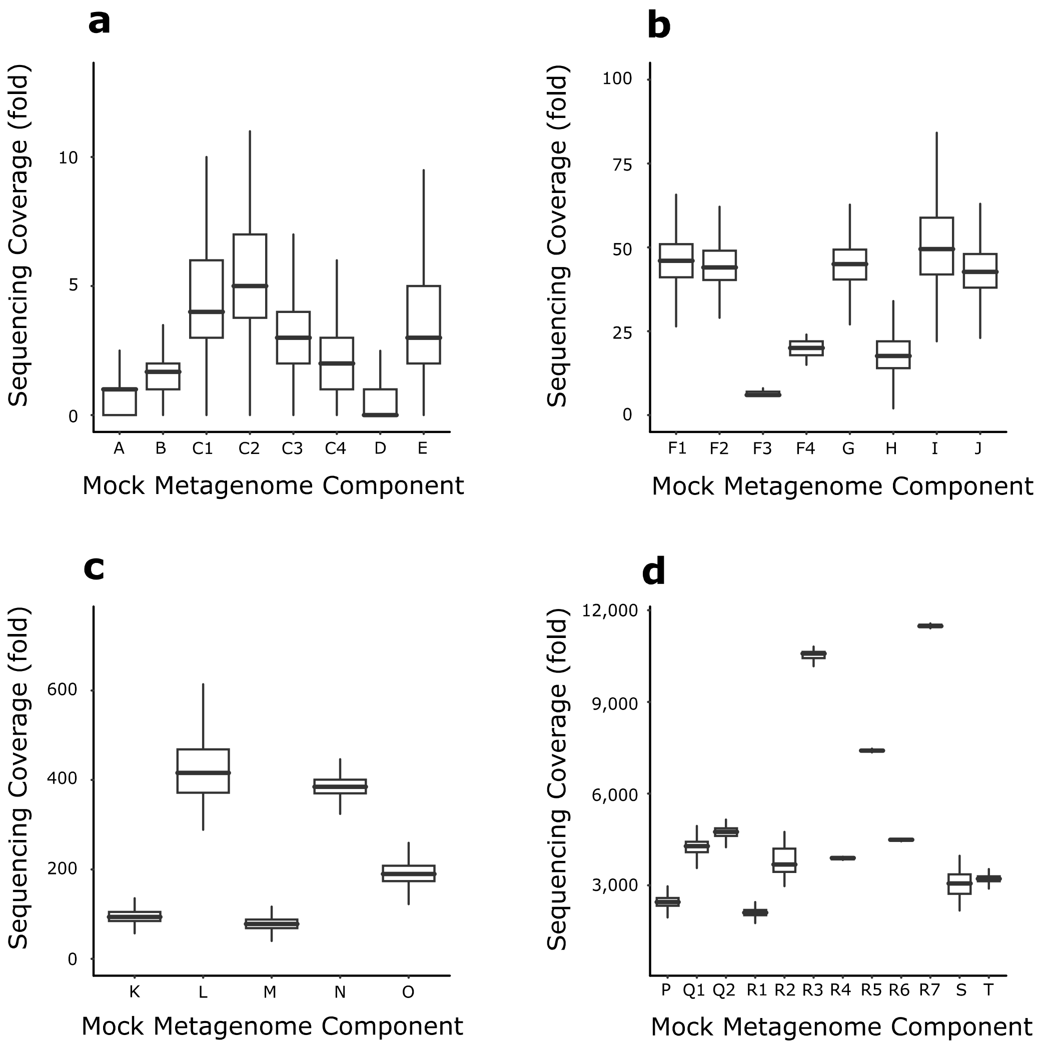

Supplementary Figure 4. Coverage distribution for metagenome samples. (a) The 0.02% composition group. A: *S. odontolytica* ,B: *E. faecalis*, C1: *D. radiodurans*-plasmid MP1,C2: *D. radiodurans*-plasmid CP1,C3: *D. radiodurans*- chromosome 1, C4: *D. radiodurans- chromosome 2*, D: *B. adolescentis*,E: *B. vulgatus.* (b) The 0.18% composition group. F1: A. baumannii- chromosome 1,F2: *A. baumannii*- chromosome 2,F3: *A. baumannii*- chromosome 3,F4: *A. baumannii*- chromosome 4, G: *N. meningitidis*,H*: L. gasseri*,I: *C. acnes*,J: *H. pylor.i* (c) Samples comprising the 1.8% composition group. K: *S. aureus*,L: *B. cereus*, M: *C. beijerinckii*,N: *P. aeruginosa*,O: *S. agalactiae.* (d) Samples comprising the 18% composition group.P: *P. gingivalis*, Q1: *R. sphaeroides*- chromosome 1,Q2: *R. sphaeroides*- chromosome 2,R1: *S. epidermidis*- chromosome 1,R2: *S. epidermidis*- chromosome 2,R3: *S. epidermidis*- chromosome 3,R4: *S. epidermidis*- chromosome 4,R5: *S. epidermidis*- chromosome 5,R6*: S. epidermidis*- chromosome 6,R7: *S. epidermidis*- chromosome 7,S: *S. mutans*,T: *E. coli.* Boxes extend from lower to upper quartiles; the median is represented by the short line within the box; whiskers extend 1.5 interquartile distances.

61 **Supplementary Figure 5**

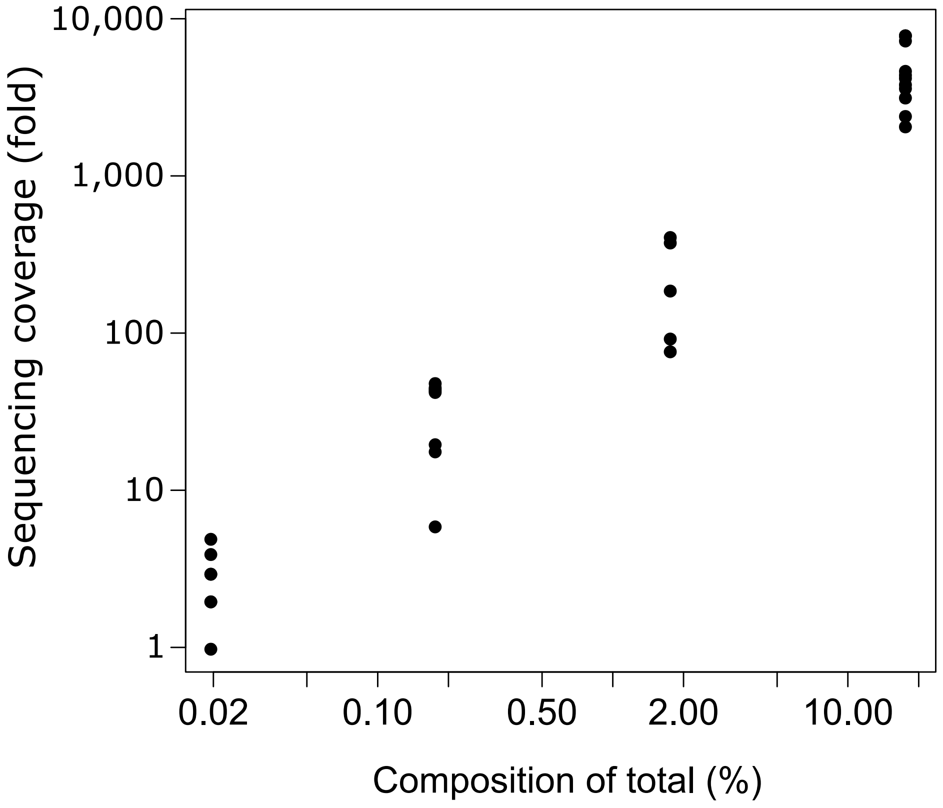

Supplementary Figure 5. Sequencing coverage vs. percent composition for the mock metagenome ATCC MSA-1003. Average sequencing coverage as mapped by minmap2 using the bacterial references listed in **Supplementary Table 1** is plotted against the expected relative composition of each bacterial species (percent of total).

Supplementary Tables

Supplementary Table 1

Sample	Strain	Composition (%)	Genome Reference	Genome size (Mb)
Acinetobacter baumannii	ATCC 17978	0.18%	CP000521	3.97
Bacillus cereus	ATCC 10987	1.80%	AE017194	5.22
Bacteroides vulgatus	ATCC 8482	0.02%	CP000139	5.16
Bifidobacterium adolescentis	ATCC 15703	0.02%	AP009256	2.09
Clostridium beijerinckii	ATCC 35702	1.80%	NZ_CP006777	6.00
Cutibacterium acnes	ATCC 11828	0.18%	CP003084	2.49
Deinococcus radiodurans	ATCC BAA-816	0.02%	AE000513<br>AE001825<br>AE001827<br>AE001826	2.65<br>0.41<br>0.05<br>0.18
Enterococcus faecalis	ATCC 47077	0.02%	NC_017316	2.74
Escherichia coli	ATCC 700926	18..0%	U00096	4.64
Helicobacter pylori	ATCC 700392	0.18%	AE000511	1.67
Lactobacillus gasseri	ATCC 33323	0.18%	CP000413	1.89
Neisseria meningitidis	ATCC BAA-335	0.18%	AE002098	2.27
Porphyromonas gingivalis	ATCC 33277	18.0%	AP009380	2.35
Pseudomonas aeruginosa	ATCC 9027	1.80%	PDLX01000000	6.34
Rhodobacter sphaeroides	ATCC 17029	18.0%	CP000577<br>NC_009050	4.37
Schaalia odontolytica	ATCC 17982	0.02%	DS264586<br>DS264585	2.39
Staphylococcus aureus	ATCC BAA-1556	1.80%	CP000255<br>CP000256<br>CP000257<br>CP000258	2.87
Staphylococcus epidermidis	ATCC 12228	18.0%	AE015929	2.50
Streptococcus agalactiae	ATCC BAA-611	1.80%	AE009948	2.16
Streptococcus mutans	ATCC 700610	18.0%	AE014133	2.03

Supplementary Table 1. Bacterial strain composition of metagenome staggered mix (ATCC® MSA-1003™).

Supplementary Table 2

SRA accession	Organism	Number of subreads (Millions)	Total bases (Gb)
**SRR12358174**	*M. musculus*	32.7	488
**SRR12371718**	*M. musculus*	31.2	466
**SRR12358173**	*Z. mays*	56.0	782
**SRR12358171**	*F. x ananassa*	21.5	428
**SRR12371721**	*R. muscosa*	24.4	346
**SRR12371723**	*R. muscosa*	22.2	318
**SRR12371724**	*R. muscosa*	29.8	419
**SRR12371725**	*R. muscosa*	23.5	328
**SRR12371726**	*R. muscosa*	24.5	343
**SRR12371727**	*R. muscosa*	22.5	316
**SRR12371722**	*R. muscosa*	22.9	323
**SRR12358172**	*R. muscosa*	22.3	319
**SRR12371719**	ATCC MSA-1003	57.9	507
**SRR12358170**	ATCC MSA-1003	57.3	512

Supplementary Table 2. Raw subread data from the 5 genome samples used for HiFi – CCS basecalling. SRA accession number, organism, number of subreads and total bases are given for each data record.

Supplementary Table 3

	Mouse	Maize	Mock Metagenome
**Mapped**	99.9%	99.99%	98.9%
**Uniquely mapped**	93.3%	96.4%	98.4%
**Unmapped**	0.1%	0.01%	1.1%

Supplementary Table 2. Mapping of HiFi reads to their references. Mouse, maize and mock metagenomes were mapped to their corresponding reference and categorized as mapped if the read aligned either uniquely (Mapping Quality Score of 60) or with an additional supplemental alignment (SAM Flag field bitcode of 0x800), uniquely mapped if it only aligned to one region of the genome with a Mapping Quality Score of 60, and unmapped if the read did not align to any region of the reference.
